# Hierarchical Clustering via Single and Complete Linkage Using Fully Homomorphic Encryption †

**DOI:** 10.3390/s24154826

**Published:** 2024-07-25

**Authors:** Lynin Sokhonn, Yun-Soo Park, Mun-Kyu Lee

**Affiliations:** 1 Department of Electrical and Computer Engineering, Inha University, Incheon 22212, Republic of Korea; lyninsokhonn@gmail.com (L.S.); yunsoo200@naver.com (Y.-S.P.); 2 Department of Computer Engineering, Inha University, Incheon 22212, Republic of Korea

**Keywords:** hierarchical clustering, single linkage, complete linkage, homomorphic encryption, CKKS scheme

## Abstract

Hierarchical clustering is a widely used data analysis technique. Typically, tools for this method operate on data in its original, readable form, raising privacy concerns when a clustering task involving sensitive data that must remain confidential is outsourced to an external server. To address this issue, we developed a method that integrates Cheon-Kim-Kim-Song homomorphic encryption (HE), allowing the clustering process to be performed without revealing the raw data. In hierarchical clustering, the two nearest clusters are repeatedly merged until the desired number of clusters is reached. The proximity of clusters is evaluated using various metrics. In this study, we considered two well-known metrics: single linkage and complete linkage. Applying HE to these methods involves sorting encrypted distances, which is a resource-intensive operation. Therefore, we propose a cooperative approach in which the data owner aids the sorting process and shares a list of data positions with a computation server. Using this list, the server can determine the clustering of the data points. The proposed approach ensures secure hierarchical clustering using single and complete linkage methods without exposing the original data.

## 1. Introduction

Clustering, also referred to as cluster analysis, is a key area of study that is particularly significant in fields such as image analysis, pattern recognition, and machine learning [1]. It serves as an exploratory data analysis technique, categorizing data into distinct groups or subsets, where elements within each subset are more similar to each other than to elements in different subsets. A primary application of clustering is assigning labels to previously unlabeled data, especially when there is no prior knowledge of their groupings [2]. Many clustering algorithms have been introduced by researchers and are frequently used in various applications. Among these, partitional and hierarchical clustering are the most popular [3,4]. The partitional approach segments a dataset directly using a specific objective function, whereas hierarchical clustering gradually creates distinct clusters. Hierarchical methods generally follow either an agglomerative path or a divisive approach. Agglomerative clustering begins with individual data points as unique clusters and develops a hierarchical structure by continuously combining these clusters in a bottom-up fashion. In contrast, divisive clustering starts with all data points in one collective cluster and breaks them down gradually [4]. Among the hierarchical techniques, agglomerative hierarchical clustering stands out for its time efficiency and enhanced computational stability [1]. In agglomerative hierarchical clustering, the two nearest clusters are consistently combined until either all points are within a single cluster or the desired number of clusters is reached [5]. The definition of “nearest” may vary. In this study, two primary distance metrics were considered: single and complete linkages. For further details, refer to Equations (1) and (2).

Clustering is a fundamental method in data analysis, but a common challenge is the use of data in its original, unencrypted form, posing risks to sensitive information. In resource-constrained environments like internet of things and sensor data applications, clustering tasks are often outsourced to external servers, necessitating robust data protection measures. Encryption offers a reliable solution to safeguard sensitive data. Particularly, homomorphic encryption (HE) [6] enables computations on encrypted data without decryption, ensuring confidentiality. HE allows mathematical operations to be performed on two ciphertexts, and the decrypted result is identical to that obtained when the operations are performed on plaintexts. The Cheon-Kim-Kim-Song (CKKS) scheme stands out in this field because it allows both addition and multiplication operations on encrypted data using an approximation-based arithmetic approach [7].

This study proposes a method that combines agglomerative hierarchical clustering using single and complete linkages with the benefits of HE. This ensures that data can be grouped appropriately without revealing their original forms. However, sorting encrypted data, a necessary step for both single- and complete-linkage clustering, poses challenges. To address this issue, we introduce a joint approach where the data owner assists in sorting and shares a list indicating the positions of the data points with the server. With this guidance, the server can accurately group data points. This approach integrates privacy preservation measures into hierarchical clustering while ensuring the confidentiality of the data involved.

## 2. Preliminaries

### 2.1. Agglomerative Hierarchical Clustering

In this paper, the term “agglomerative hierarchical clustering” will be referred to simply as “hierarchical clustering”. The process of hierarchical clustering is outlined in the following steps.

Step 1: First, each data point is regarded as its own separate cluster, resulting in a total of n distinct clusters.

Step 2: As the procedure progresses, the two nearest clusters are combined into one. For instance, given a set of clusters labeled as $\left\{C_{1},\;C_{2},\ldots ,\;C_{n}\right\}$, when the two closest clusters $C_{i}$ and $C_{j}$ are determined, they are merged to create a new cluster, $C_{\mathrm{ij}}$.

Step 3: After merging $C_{i}$ and $C_{j},$ they are replaced in the set by $C_{\mathrm{ij}}$, reducing the number of clusters by one.

This merging process (Steps 2 and 3) is repeated until a single comprehensive cluster is formed, yielding a sequence of nested clusters. If necessary, merging can stop once a specified number of clusters k is reached.

In Step 2 of the clustering process, the proximity between two clusters can be determined using several methods. In this study, two main distance measurement methods were studied: single linkage and complete linkage.

For two clusters $C_{i}$ and $C_{j}$, the single linkage distance $D(C_{i},\;C_{j})$ is defined as the shortest Euclidean distance between a point in $C_{i}$ and a point in $C_{j}$. This is expressed as follows:

(1) $$D\left(C_{i},\;C_{j}\right)=min\left\{\left\|x-y\right\|\;|\;x\in C_{i},\;y\in C_{j}\right\},$$

**Figure 1:**
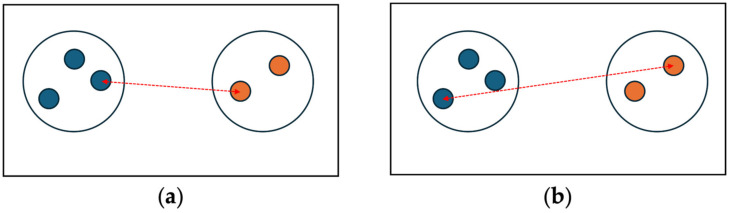
Distance between two clusters defined by (**a**) single linkage and (**b**) complete linkage.

A single linkage distance can be visualized as shown in Figure 1a. On the other hand, the complete linkage distance between two clusters is determined by the longest Euclidean distance among all pairs of points, as shown in

(2) $$D\left(C_{i},C_{j}\right)=max\left\{\left\|x-y\right\|\;|\;x\in C_{i},y\in C_{j}\right\},$$

The visualization of the distance between two clusters defined by complete linkage is illustrated in Figure 1b.

In Equations (1) and (2), $\left\|x-y\right\|$ denotes the Euclidean distance between *x* and *y*. These calculated distances are then used to construct a distance matrix, where the element at the ith row and jth column represents the distance between $C_{i}$ and $C_{j}$. During Step 3, when two clusters merge, the distance matrix is updated by recomputing the distances from the newly combined cluster to the others. While the distances from the merged cluster to the others must be updated with every merge, the distances amongst the other clusters remain unchanged [5].

### 2.2. Homomorphic Encryption

Homomorphic encryption (HE) [6] preserves the algebraic structure, allowing computations on encrypted data without requiring decryption. Fully homomorphic encryption supports an unlimited number of additions and multiplications, which are core operations for deriving more complex functions [7]. While schemes such as BGV [8,9] and BFV [8,10] primarily support operations on integers, the CKKS scheme broadens this scope to include real and complex numbers [11]. The CKKS scheme supports approximate operations, crucial for statistical analyses and machine learning.

The “Homomorphic Encryption for Arithmetic of Approximate Numbers” (HEaaN) is a specialized library that implements the CKKS scheme, offering features like key generation, encryption, decryption, and homomorphic operations [12]. In the CKKS scheme, data are represented as polynomials, which are divided into components referred to as slots. Each slot can independently hold a number, either complex or real, enabling parallel operations. In this study, we represent a plaintext vector A as $\langle A\rangle$ once encrypted. Arrays containing multiple elements, whether ciphertexts or plaintexts, are denoted by parentheses. The operation ‘mult()′ represents element-wise multiplication between two ciphertexts or between a ciphertext and a plaintext. Similarly, ′add()′ and ′sub()′ denote element-wise addition and subtraction, respectively. Additionally, a ciphertext can be shifted either to the left or the right by a specified number of rotations using the ′left_rotate()’ and ‘right_rotate()′ functions.

### 2.3. Privacy-Preserving Clustering

Recent advancements in cryptographic methods have spurred the development of privacy-preserving clustering algorithms. Much of this research has focused on centroid-based clustering, employing techniques such as HE, secure multiparty computation, or a combination thereof, to safeguard data privacy during clustering operations [13,14,15,16,17,18,19,20].

Additionally, density-based clustering methods have been adapted for encrypted environments to ensure privacy, enabling data grouping without direct access to raw data [21,22,23].

A smaller subset of studies has investigated hierarchical clustering within privacy-preserving frameworks. Meng et al. [24], for instance, integrated HE and multiparty computation to facilitate hierarchical clustering while maintaining data confidentiality throughout various stages of data processing.

Our research contributes to this field by implementing hierarchical clustering using the CKKS scheme of HE, an approach that has been relatively less widely explored. This methodology allows us to perform hierarchical clustering directly on encrypted data, ensuring privacy throughout the entire data analysis process.

## 3. Proposed Approach

The goal of this study was to perform hierarchical clustering using HE. The proposed approach closely follows the standard clustering process. However, in Step 3, where distances between initially separate clusters remain unchanged but need updating in the distance matrix, we opted for sorting instead of recomputation.

**Figure 2:**
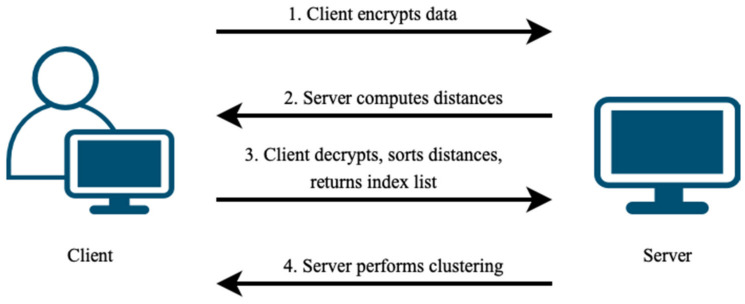
Client–server process flow for handling and clustering encrypted data.

With HE, data are represented as ciphertext blocks. The sorting function in HEaaN, though powerful, is computationally intensive and sorts only the values within ciphertext slots without preserving original index positions. When merging clusters, knowing both the distances and the original cluster indices is crucial. Therefore, we propose a collaborative approach involving the data owner. The data owner assists in sorting distances and provides the original index positions of the initial single clusters (data points). Since sorting alters the original positions, sharing these post-sorted positions does not compromise the confidentiality of encrypted data. The process begins with the client (the data owner) encrypting the data and transmitting it to the server for distance calculation. The server then sends intermediate results back to the client. After decrypting and sorting the distances, the client sends the sorted indices corresponding to these distances back to the server for clustering. The process flow is illustrated in Figure 2.

Suppose we have a ciphertext containing n data points, where each data point has *N* features (assuming n and N are powers of two for simplicity). The ciphertext is rotated to allow distance computation between all possible combinations of individual data points, as detailed in Algorithm 1. We denote the i-th data point as $P_{i-1}=\left(P_{i-1}^{0},\;P_{i-1}^{1},\;\ldots ,\;P_{i-1}^{N-1}\right)$.
**Algorithm 1:** Computation of DistanceList**Input:**   A ciphertext $\langle X\rangle =\langle \left(P_{0}^{0},\;P_{0}^{1},\;\ldots ,\;P_{0}^{N-1},\;P_{1}^{0},\;P_{1}^{1},\;\ldots ,\;P_{1}^{N-1},\ldots ,P_{n-1}^{0},\;P_{n-1}^{1},\;\ldots ,\;P_{n-1}^{N-1}\right)\rangle$ **Output:**   $DistanceList=(\langle D_{0}\rangle ,\;\;\langle D_{1}\rangle ,\;\ldots ,\;\langle D_{\frac{n}{2}-1}\rangle )$ 1: **for** i=0 to $\frac{n}{2}-1$ **do**
$\left\{0\leq i\leq \frac{n}{2}-1\right\}$ 2:     $\langle R_{i}\rangle \;\leftarrow \;left\_rotate\left(\langle X\rangle ,\;\;(i+1)\cdot N\right)$ 3:     $\langle D_{i}\rangle \;\leftarrow \;D\left(\langle X\rangle ,\;\;\langle R_{i}\rangle \right)$ 4:     append $\langle D_{i}\rangle$ to DistanceList 5: **end for** 6: **return** DistanceList


Algorithm 2 outlines the process of computing the Euclidean distance for each pair of data points. The output of this algorithm is the nN-dimensional ciphertext vector $\langle \left({\left\|P_{0}-P_{\left(1+i\right)\;mod\;n}\right\|}^{2},0,\;0,\;\ldots ,\;0,\;{\left\|P_{1}-P_{\left(2+i\right)\;mod\;n}\right\|}^{2},\;0,\;0,\;\ldots ,\;0,\;\ldots ,{\left\|P_{n-1}-P_{\left(n+i\right)\;mod\;n}\right\|}^{2}\right)\rangle$, where 0, 0, …, 0 represents the sequence of N−1 zeros.
**Algorithm 2:** $D\left(\langle X\rangle ,\;\langle R_{i}\rangle \right)$: Computation of Euclidean distance**Input:**   Ciphertext $\langle X\rangle$, $\langle R_{i}\rangle$: the i+1th rotation of $\langle X\rangle$ **Output:**   Euclidean distance list $\langle D_{i}\rangle$ 1: $\langle D_{i}\rangle \;\leftarrow \;sub\left(\langle X\rangle ,\;\;\langle R_{i}\rangle \right)$ 2: $\langle D_{i}\rangle \;\leftarrow \;mult\left(\langle D_{i}\rangle ,\;\;\langle D_{i}\rangle \right)$ 3: **for** index=0 to $\left(\log_{2}N-1\right)$ **do** $\left\{0\leq index\leq \log_{2}N-1\right\}$ 4:     $\langle d\rangle \;\leftarrow \;left\_rotate(\langle D_{i}\rangle ,\;\;2^{index})$ 5:     $\langle D_{i}\rangle \;\leftarrow \;add\left(\langle D_{i}\rangle ,\;\;\langle d\rangle \right)$ 6: **end for** 7: **initialize** T as a plaintext of 0s with length n·N 8: **for** index=0 to $\left(n-1\right)$ **do** $\left\{0\leq index\leq n-1\right\}$ 9:     $T\left[index\cdot N\right]\;\leftarrow \;1$ 10: **end for** 11:  $\langle D_{i}\rangle \;\leftarrow \;mult\left(\langle D_{i}\rangle ,\;\;T\right)$ 12: **return** $\langle D_{i}\rangle$


In the computation of Euclidean distance, the result is a list of squared distances, not the distances themselves. Since the actual distance values are unnecessary and squared Euclidean distances increase monotonically with Euclidean distances, the list of squared distances is sufficient for subsequent sorting tasks. After computing DistanceList, it is forwarded to the data owner for sorting.

The data owner sorts all distances within DistanceList and provides the server with the corresponding indices that match the sorted distances. Upon receiving these indices, the server begins with the clustering process based on the sorted index list returned by the data owner.

### 3.1. Single Linkage

Algorithm 3 presents the clustering procedure using single linkage, employing union-find operations to manage disjoint sets (i.e., clusters) of data points. In lines 4–20, the algorithm iteratively processes each pair from the sorted index pairs until the number of clusters reduces to the specified number, k. The algorithm begins by examining the first pair representing the two closest data points. The Find function, defined in lines 28–33, determines the cluster identifier, or the root node of the set tree to which the input element belongs. If two elements in a pair share the same root, indicating that they are already part of the same cluster, the pair is discarded. Conversely, if the roots are different, as checked in line 8, the two elements are combined to form a new cluster. Lines 12 and 13 update the root nodes of the newly formed cluster to reflect the new cluster identifiers. The original clusters that were merged are then cleared, indicating that their elements are part of the new cluster. Following the merging, the processed pair is removed from the list, and the algorithm proceeds to the next closest pair. This procedure is repeated until the desired number of clusters k is reached.
**Algorithm 3:** SingleLinkage(Pairs, d, k): Clustering via single linkage**Input:** Pairs: a list of sorted index pairs, d: number of data points, k: desired number of clusters **Output:** FinalClusters: a list of clusters 1: $ClusterMap\;\leftarrow \;\left\{i\to i\;|\;i\in Z_{d}\right\}$ 2: $Clusters\;\leftarrow \;\left\{\left[i\right]\;|\;i\in Z_{d}\right\}$ 3: c ← d, n ← d 4: **while** c>k **do** 5:         $\left(i,\;j\right)\;\leftarrow \;Pairs\left[0\right]$ 6:         root1 ← Find(ClusterMap, i) 7:         root2 ← Find(ClusterMap, j) 8:         **if** root1≠root2 **then** 9:             MergedCluster ← Clusters[root1]∪Clusters[root2] 10:              append MergedCluster to Clusters 11:              $ClusterMap\left[n\right]\;\leftarrow \;n$ 12:              $ClusterMap\left[root1\right]\leftarrow \;n$ 13:              $ClusterMap\left[root2\right]\leftarrow \;n$ 14:              empty Clusters[root1] 15:              empty Clusters[root2] 16:              c ← c−1 17:              n ← n+1 18:         **end if** 19:         Pairs ← Pairs[1:] 20: **end while** 21: **initialize** FinalClusters as an empty list 22: **for** each cluster in Clusters **do** 23:         **if**
cluster is not empty **then** 24:              append cluster to FinalClusters 25:         **end if** 26: **end for** 27: **return**
FinalClusters 28: **function**
Find (ClusterMap, i) 29:         **while**
$ClusterMap\left[i\right]\neq i$ **do** 30:              i ← ClusterMap[i] 31:         **end while** 32:         **return**
i 33: **end function**

**Figure 3:**
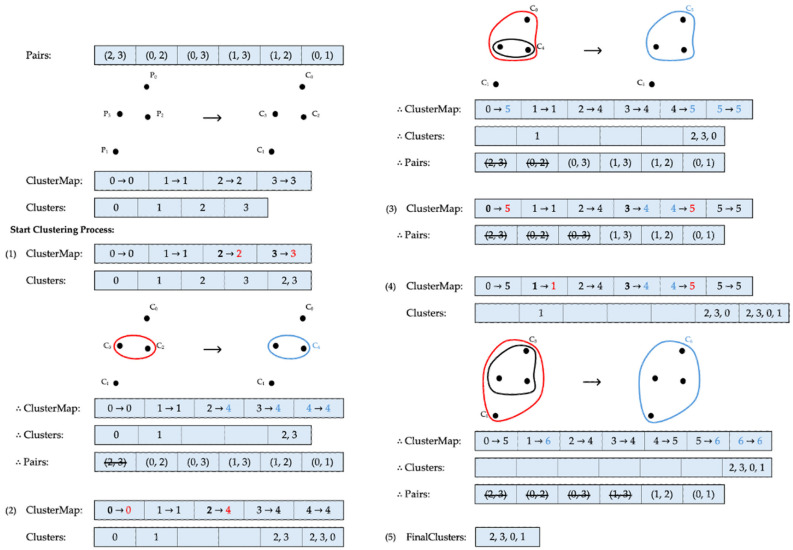
Clustering via single linkage. Cluster identifiers before and after each update are marked in red and blue, respectively.

Figure 3 illustrates an example of the clustering process using the single linkage method, where k=1. Starting with four data points, each data point initially forms a separate cluster. The roots of these clusters point to identifiers that match the cluster labels. According to the Pairs list provided by the client, the first elements to be processed are $C_{2}$ and $C_{3}$. Because both elements point to different roots, they qualify for merging. This new combination is then added to the existing Clusters list, and the roots of $C_{2}$ and $C_{3}$ in ClusterMap are updated to a new cluster identifier, $C_{4}$. Following this, the elements that have already been merged are cleared from their previous positions in Clusters, and the processed pair is completely removed from the index pair list. After this merging process, three clusters remain. The next pair to be considered is $C_{0}$ and $C_{2}$. Although the root of $C_{2}$ has changed to $C_{4}$, the pair still points to different roots, qualifying them for merging. All elements of $C_{4}$ and $C_{0}$ are then added to Clusters, creating another cluster identifier, $C_{5}$, which updates the identifiers of the related clusters accordingly. After merging, the now irrelevant clusters are cleared from the cluster list, and the merged pair is removed. At this stage, two clusters, $C_{1}$ and $C_{5}$, remain. The next elements to be processed are $C_{0}$ and $C_{3}$. However, because of the previous merging, $C_{0}$ and $C_{3}$ have already formed one cluster, sharing the same root. Therefore, this pair is skipped, and the algorithm moves on to the next pair, $C_{1}$ and $C_{3}$. This pair can be combined, as they have separate roots, resulting in the creation of $C_{6}$ as a new cluster identifier. After following similar processing steps as in the previous merges, only two pairs remain in the Pairs list. Since these remaining pairs belong to the same cluster and every data point has now become part of one cluster, the procedure concludes. Consequently, all individual clusters, $C_{2},$ $C_{3}$, $C_{0}$, and $C_{1}$, are merged into one final cluster, as shown in Figure 3.

### 3.2. Complete Linkage

**Figure 4:**
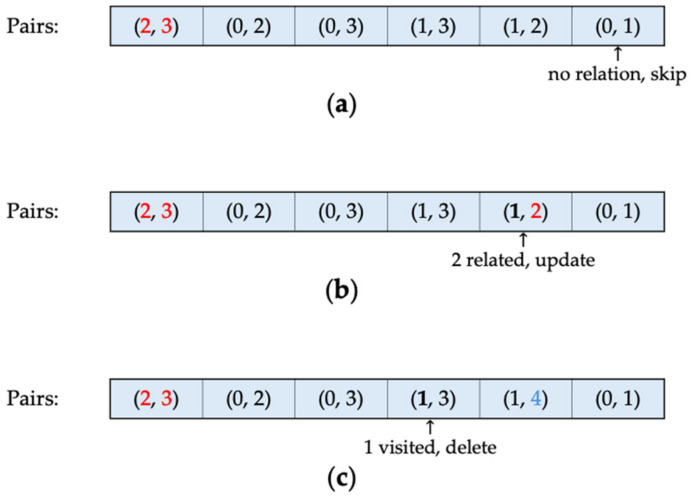
Case where the pair in focus (**a**) has no relation to the formed cluster, (**b**) is related to the formed cluster, and (**c**) defines a shorter distance between two clusters.

Algorithm 4 demonstrates the clustering procedure using complete linkage. Similar to single linkage, the closest clusters are merged; however, the key difference lies in the distance used to represent the two clusters. The complete linkage distance is defined as the maximum distance between any two points in the clusters, as outlined in Equation (2). The primary objective of Algorithm 4 is to identify the closest clusters with the maximum inter-cluster distance, using a sorted index pair list. The DistanceUpdate function, defined in lines 21–56, accomplishes this by identifying the pair with the longest distance, removing pairs with shorter distances in the newly formed cluster, and updating the cluster identifiers to reflect the current state of cluster formation. When a list of sorted index pairs is provided, the first pair represents the shortest distance among all combinations of points or single clusters. Consequently, the first pair is combined to form a new cluster, and the cluster identifiers are updated. Figure 4 illustrates various scenarios in which cluster pairs are handled following the DistanceUpdate function. Initially, when an index list is presented by the client in ascending order according to their distances, the first two cluster identifiers—2 and 3, representing the closest distance—are merged to create a new cluster with a new identifier, 4.
**Algorithm 4:** CompleteLinkage(Pairs, d, k): Clustering via complete linkage**Input:**   Pairs: a list of sorted index pairs, d: number of data points, k: desired number of clusters **Output:**   FinalClusters: a list of clusters 1: $Clusters\;\leftarrow \;\left\{\left[i\right]\;|\;i\in Z_{d}\right\}$ 2: c ← d, n ← d 3: **while** c>k **do** 4:          $\left(i,\;j\right)\;\leftarrow \;Pairs\left[0\right]$ 5:        MergedCluster← Clusters[i]∪Clusters[j] 6:        append MergedCluster to Clusters 7:        empty Clusters[i] 8:        empty Clusters[j] 9:        Pairs ← DistanceUpdate(Pairs,n) 10:          Pairs ← Pairs[1:] 11:          c ← c−1 12:          n ← n+1 13: **end while** 14: **initialize** FinalClusters as an empty list 15: **for** each cluster in Clusters **do** 16:          **if**
cluster is not empty **then** 17:              append cluster to FinalClusters 18:          **end if** 19: **end for** 20: **return**
FinalClusters 21: **function**
DistanceUpdate(Pairs,  n) 22:          $\left(i,\;j\right)\;\leftarrow \;Pairs[0]$ 23:          l ← length of Pairs list 24:          **if** l=1 **then** 25:              Pairs[0]←(n, n) 26:              **return** Pairs 27:          **else** 28:                 **initialize** Deleted as an empty list 29:                 **initialize** Visited as a list of 0s with length n 30:                 **for** x=l−1 to 1 **do** $\left\{l-1\geq index\geq 1\right\}$ 31:                     $\left(i^{\prime },\;j^{\prime })\leftarrow Pairs[x\right]$ 32:                     **if** $i^{\prime }=i$ or $i^{\prime }=j$ **then** 33:                           **if** $Visited\left[j^{\prime }\right]=1$ **then** 34:                                  append x to Deleted 35:                           **else** 36:                                  $Pairs[x]\leftarrow (n,\;j^{\prime })$ 37:                                  $Visited[j^{\prime }]\leftarrow 1$ 38:                           **end if** 39:                     **else if** $j^{\prime }=i$ or $j^{\prime }=j$ **then** 40:                           **if** $Visited\left[i^{\prime }\right]=1$ **then** 41:                                  append x to Deleted 42:                           **else** 43:                                  $Pairs[x]\leftarrow (i^{\prime },\;n)$ 44:                                  $Visited[i^{\prime }]\leftarrow 1$ 45:                           **end if** 46:                     **end if** 47:                 **end for** 48:                 **initialize** NewPairs as an empty list 49:                 **for** index=0 to (l−1) **do** $\left\{0\leq index\leq l-1\right\}$ 50:                     **if** index not in Deleted **then** 51:                           append Pairs[index] to NewPairs 52:                     **end if** 53:                 **end for** 54:                 **return** NewPairs 55:          **end if** 56: **end function**

As a non-single cluster now exists, the index pair list must be adjusted; otherwise, the next pair to be merged in ascending order will not represent the maximum distance of the cluster. This adjustment involves searching the list backward to identify pairs related to the newly formed cluster. Starting from the end of the list, if a pair is not related to the formed cluster, this indicates that the pair is outside the cluster and should remain unchanged during the current step. Figure 4a shows the case where the longest distance, indicated by pairs 0 and 1, corresponds to the cluster identifiers that are not connected to the merged identifiers 2 and 3.

Conversely, if a pair is related to the cluster—meaning one of the elements in the pair belongs to the previously formed cluster—the first encounter of such a relation indicates that the pair represents the maximum distance from a single cluster to the formed cluster. Consequently, the cluster identifier is updated, and its counterpart element is marked as visited. In Figure 4b, the second-longest distance corresponds to the distance between clusters 1 and 2, where cluster 2 already belongs to a previously merged cluster. This represents the longest distance between Cluster 1 and Cluster 4. Therefore, Cluster 2, no longer a single cluster but part of Cluster 4, updates its identifiers to four, transforming pair (1, 2) to (1, 4).

As the process advances towards the front of the list, encountering another pair related to the formed cluster where the paired element, initially a single cluster, has been encountered previously, indicates that its distance is not the maximum distance from the formed cluster to the single-clustered element. Consequently, the pair is marked for deletion from the list. In Figure 4c, because the maximum distance between Clusters 1 and 4 has already been identified, any pair representing the distance from Cluster 1 to other elements of Cluster 4 is removed, as it would indicate shorter distances between the two clusters. Once all pairs are processed and the search returns to the list’s front, the remaining pairs are valid, representing the longest distance from the previously formed cluster to every other cluster. The clustering procedure proceeds from the front, followed by another backward search.

In Algorithm 4, the complete linkage clustering begins by sequentially processing each pair of data points or cluster identifiers, starting with the closest. The two clusters are merged into a new cluster, as shown in line 5. The original data points belonging to a cluster are no longer considered single clusters and must be repositioned to reflect their new identifiers. After merging, the cluster identifier and index pair list are updated using the DistanceUpdate function. This function compiles a new index pair list, excluding those pairs marked for deletion, thereby maintaining an updated and relevant list of pairs for further processing. The initial pair processed for clustering is then removed, and the clustering procedure is repeated until the number of remaining clusters is reduced to k. At the end of the process, FinalClusters includes only valid clusters that are not empty.

**Figure 5:**
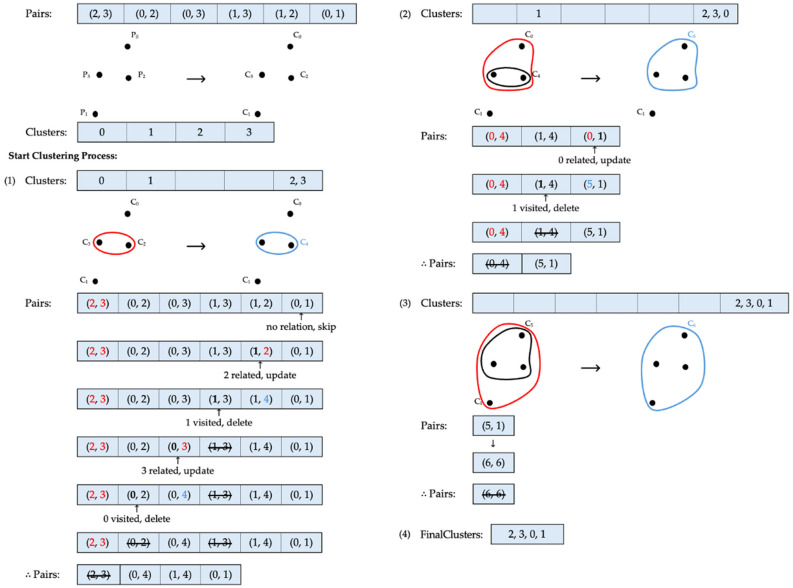
Clustering via complete linkage. Cluster identifiers before and after each update are marked in red and blue, respectively.

In Figure 5, Clusters is a list used to store each cluster as the procedure progresses. The process begins with the Pairs list provided by the client. According to this list, $C_{2}$ and $C_{3}$ are identified as the closest single clusters to be merged, forming a new cluster, $C_{4}$. After merging, the new combination of $C_{2}$ and $C_{3}$ is added to Clusters, and their previous separate cluster positions are emptied. The Pairs list is then scanned backward using the DistanceUpdate function mentioned in Algorithm 4 to update pairs connected to $C_{2}$ and $C_{3}$, and to remove pairs representing shorter Euclidean distances. Since $C_{0}$ and $C_{1}$ have no relation to the newly formed cluster, this pair can be safely skipped. Next, the pair $C_{1}$ and $C_{2}$ is considered. Although $C_{2}$ already belongs to the new cluster, this is the first encounter with $C_{1}$. Therefore, the cluster identifier of $C_{2}$ is updated to its new cluster, $C_{4}$, and $C_{1}$ is marked as visited. This pair represents the maximum distance from $C_{4}$ to $C_{1}$. The next pair to be considered is $C_{1}$ and $C_{3}$. Since $C_{3}$ belongs to $C_{4}$, this pair represents the distance from $C_{4}$ to another single cluster, which cannot be the maximum distance, given that the list is sorted in ascending order. Thus, the pair $C_{1}$ and $C_{3}$ is removed from the list. Following this, $C_{0}$ and $C_{3}$ are processed. Similar to the previous case, since $C_{3}$ is part of $C_{4}$ and $C_{0}$ has not been visited before, $C_{3}$ is updated to its new cluster identifier, $C_{4}$, and $C_{0}$ is marked as visited. For the pair $C_{0}$ and $C_{2}$, since $C_{2}$ is already part of the formed cluster and $C_{0}$ has been visited previously, this pair does not represent the maximum distance from $C_{4}$ to $C_{0}$. Therefore, this pair is also removed from the Pairs list. Now that all pairs in the list have been processed, the first pair that formed the cluster is omitted from the list. At this stage, three clusters remain after the initial merge. Using the updated Pairs list, the next closest pair identified is $C_{0}$ and $C_{4}$, forming a new cluster identifier, $C_{5}$. All elements from $C_{4}$ and $C_{0}$ are added to the cluster list, and their previous positions are cleared from Clusters. Starting the search from the back of the Pairs list, $C_{0}$ and $C_{1}$ are the first elements to be processed. Since $C_{0}$ now belongs to the new cluster $C_{5}$, and this is the first encounter with $C_{1}$, the distance represented by this pair must be the maximum distance between $C_{5}$ and $C_{1}$. Thus, the cluster identifier of $C_{0}$ is updated to $C_{5}$, and $C_{1}$ is marked as visited. In the next pair, $C_{1}$ and $C_{4}$, since $C_{4}$ is not a single cluster anymore and $C_{1}$ has already been visited, this pair is discarded. After this step, the merged pair, $C_{0}$ and $C_{4}$, is removed from Pairs, leaving only $C_{5}$ and $C_{1}$. With only one pair left to be combined, after merging, their cluster identifiers are updated to the same identifier. As a result, all four data points now become part of one final cluster.

## 4. Implementation

**Figure 6:**
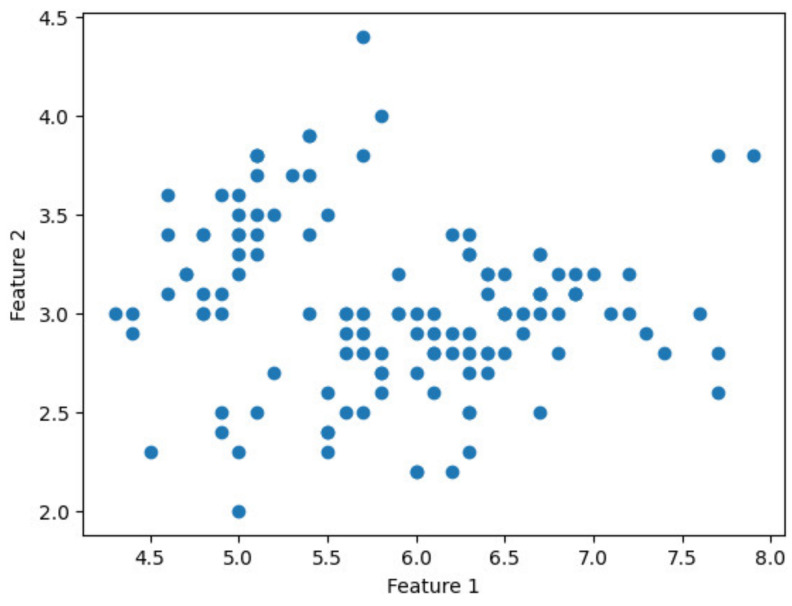
Scatter plot of feature 1 vs. feature 2 before clustering.

To verify the feasibility of the proposed approach, it was implemented on the Iris dataset [25] using the HEaaN library. The Iris dataset, widely recognized in the fields of machine learning and data analysis, comprises 150 data points, each with four features: sepal length, sepal width, petal length, and petal width. Due to the requirement that ciphertext dimension be a power of two, only 128 data points were used for the implementation. The experiment was conducted using the FGb parameter preset in HEaaN and executed on an NVIDIA TITAN RTX GPU featuring 4608 CUDA cores. The GPU was sourced from NVIDIA Corporation, Santa Clara, CA, USA. The sort function in Python 3.8.16 was employed for client-side sorting. Figure 6 presents a scatter plot illustrating the distribution of the data points in the Iris dataset before clustering, specifically using features 1 (sepal length) and 2 (sepal width).

**Figure 7:**
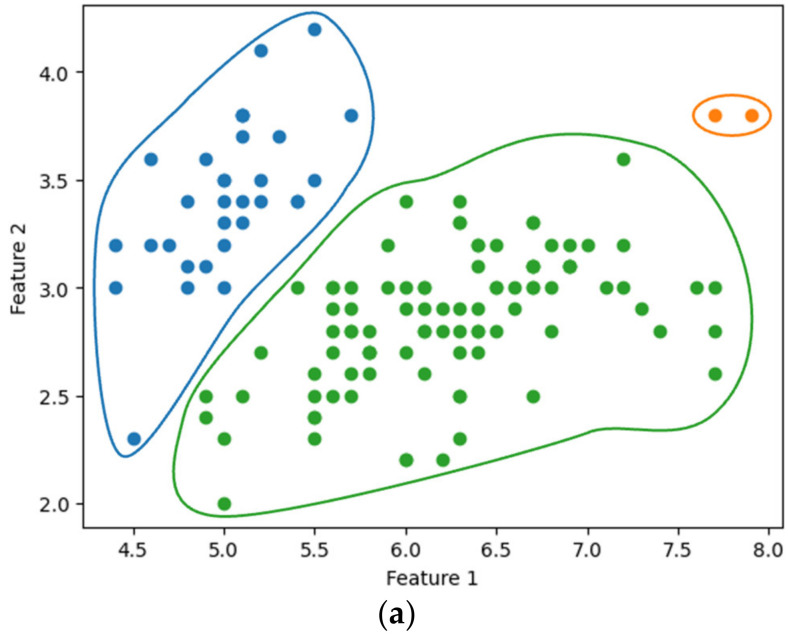

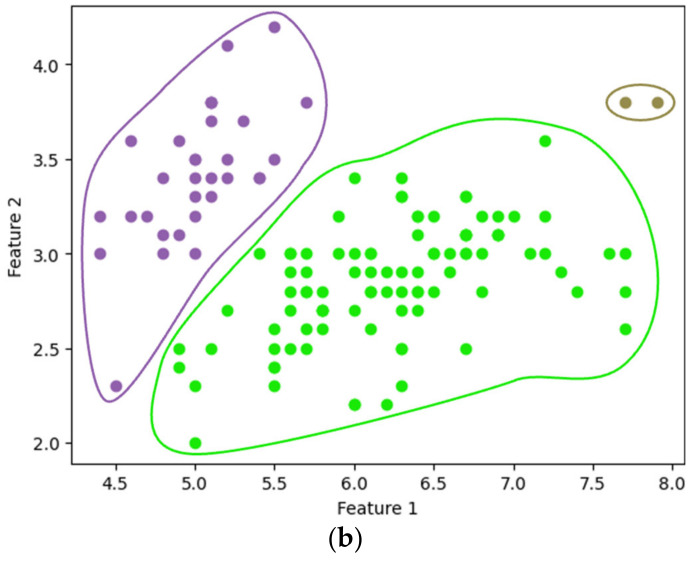
Single linkage clustering with k=3: using (**a**) SciPy and (**b**) the proposed method.

**Figure 8:**
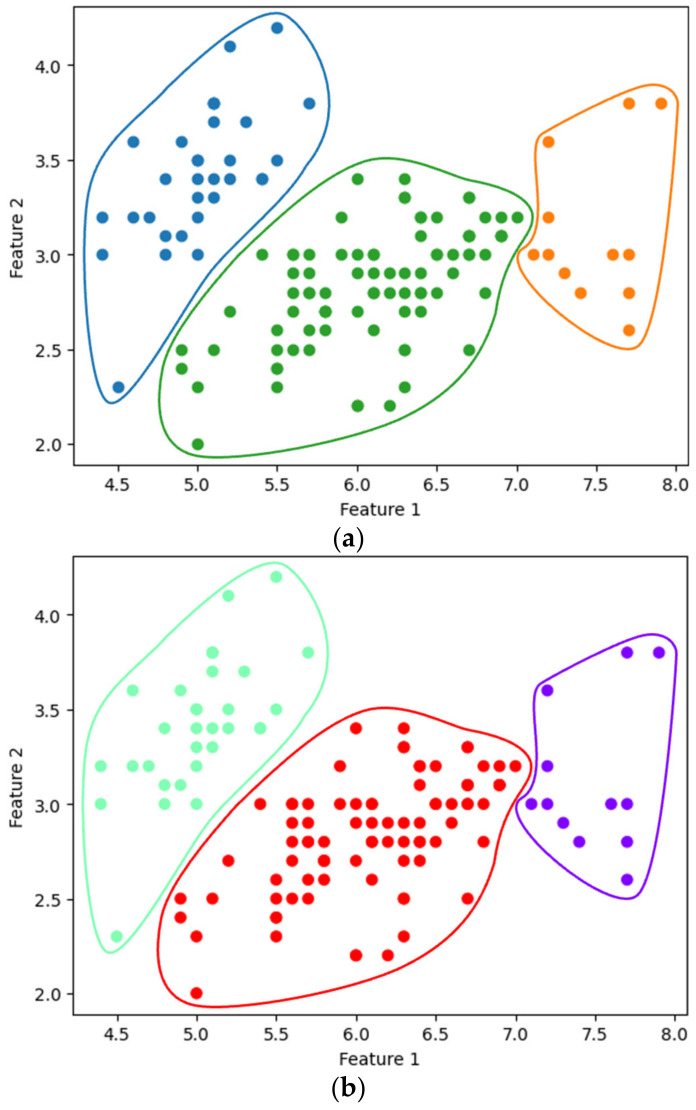
Complete linkage clustering with k=3: using (**a**) SciPy and (**b**) the proposed method.

Figure 7a,b show the single-linkage clustering results for three clusters (k=3), obtained using the existing SciPy library and our method, respectively. The entire process of our method, from data encryption to clustering, was completed in approximately 2.085 s. Similarly, Figure 8 compares the results of complete linkage clustering for the three clusters. Figure 8a displays the outcome from the SciPy library, while Figure 8b presents the results obtained using our method, completed in 2.918 s. Our clustering approach yielded results consistent with those produced by the widely used SciPy library.

Further testing of the proposed clustering method was conducted using Scikit-learn to evaluate its performance across various desired numbers of clusters, ranging from 2 to 10 clusters. For each configuration, the method was iterated 10 times with 128 data points randomly sampled from the Iris dataset. The evaluation focused on detecting misassigned points in comparison to clusters generated by the Scikit-learn library. Consistency is defined as the absence of misassigned points across all iterations for each number of clusters. Throughout these tests, no misassigned points were observed in either single or complete linkage clustering, demonstrating the reliability and consistency of the proposed method across different numbers of clusters. This consistency highlights the suitability of our approach for diverse clustering scenarios compared to existing methods.

**Figure 9:**
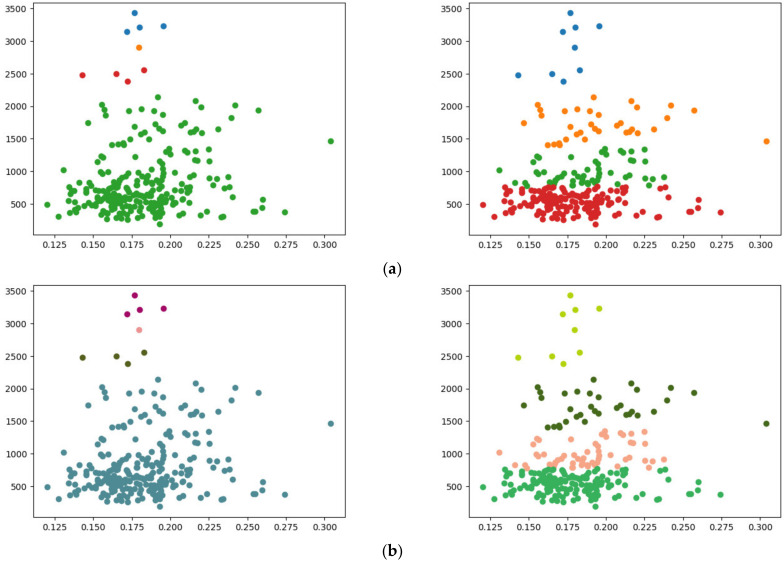
Comparison of clustering conducted on the Breast Cancer Wisconsin (diagnostic) dataset: (**a**) SciPy results for single (**left**) and complete (**right**) linkage; (**b**) results from our method for single (**left**) and complete (**right**) linkage.

In addition to the Iris dataset, experiments were also conducted on other datasets, varying the numbers of features, data points, and clusters. One such dataset is the Breast Cancer Wisconsin (Diagnostic) dataset, which comprises 569 instances and 30 features [26]. For this experiment, 256 data points with eight features were sampled. Our clustering process, employing both single and complete linkage methods, yielded the following results: for k=4, single linkage took approximately 3.392 s, while complete linkage took an average of 7.875 s. These results are compared in Figure 9, where (a) shows the clustering outcomes using the SciPy library and (b) illustrates the results from our approach. Our method consistently produced results similar to those from SciPy for both linkage methods.

**Figure 10:**
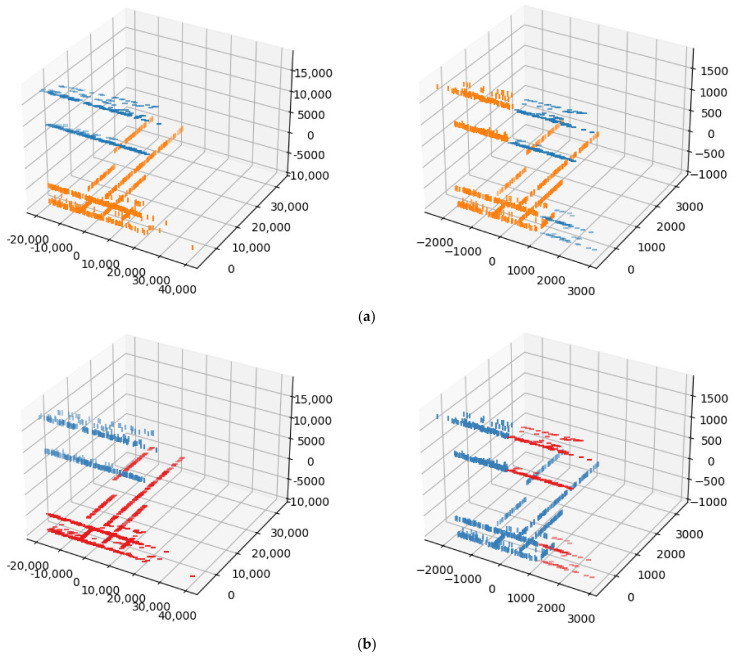
Comparisons of clustering conducted on the UGRansome dataset: (**a**) SciPy results for single (**left**) and complete (**right**) linkage; (**b**) results from our method for single (**left**) and complete (**right**) linkage.

Another set of tests was conducted on the UGRansome dataset [27], a network traffic dataset. We sampled 1024 instances, each with three features. To align with our clustering algorithm’s requirement of ciphertext values being powers of two, we added zero-padding to include a fourth feature. Figure 10 presents the comparison of the clustering results for k=2 clusters. Figure 10a displays the results from SciPy version 1.10.1, while Figure 10b shows the results obtained from our method. For our method, single linkage took 13.561 s, and complete linkage took 268.567 s.

Notably, a slight difference appeared in the clustering results obtained from the two methods. For instance, in the complete linkage clustering, data points at indexes 532, 832, and 970 were clustered into the second cluster (the orange cluster in Figure 10a), whereas they were assigned to the first cluster using our method (the red cluster in Figure 10b). This discrepancy is discussed further in Section 5.

## 5. Discussion

Table 1 compares our approach with prior studies on privacy-preserving clustering, outlining the methodologies and privacy-preserving techniques employed in each study [28]. Previous research has predominantly focused on centroid-based clustering, specifically the k-means algorithm [13,14,15,16,17,18,19,20]. Additionally, density-based clustering methods, such as mean shift [21] and DBSCAN [22,23], have been explored. Hierarchical clustering has also been investigated, particularly through the integration of HE and secure multi-party computation (MPC) [24]. In contrast, our study focuses exclusively on enhancing hierarchical clustering via HE.

The CKKS scheme is based on approximation, which inherently introduces precision errors [29]. For instance, in our experiments with the UGRansome dataset, these errors led to slight discrepancies in the computed distances, causing some data points to be clustered differently than in SciPy’s implementation. Despite these variations, particularly noticeable at small distance values, our method aligns with the fundamental principles of both single and complete linkage clustering.

## 6. Conclusions

Traditional hierarchical clustering methods often operate directly on raw data, which can expose sensitive information and compromise data privacy and security. In contrast, the use of HE ensures data privacy and reduces the risk of information leakage, providing a solution for preserving sensitive information during data analysis. By leveraging the HEaaN library, this study demonstrated a methodology for performing agglomerative hierarchical clustering in a privacy-preserving manner using both single and complete linkage methods.

Our experiment with various datasets served as a practical demonstration of the feasibility and effectiveness of the proposed approach. Both the single and complete linkage methods produced results that closely aligned with outcomes derived from widely used libraries that perform computations in plaintext. This alignment underscores the validity and reliability of the proposed clustering approach and its potential for real-world applications.

## Figures and Tables

**Table 1:** Comparison of privacy-preserving clustering approaches.

Clustering Type	Privacy Technique	Paper
Centroid-based	HE	[13,14,15,16]
	MPC	[17,18]
	HE + MPC	[19]
	HE and MPC (two protocols)	[20]
Density-based	HE	[21]
	MPC	[22,23]
Hierarchical	HE + MPC	[24]
	HE	Our work

## Data Availability

Data is contained within the article.
